# Questing abundance of adult taiga ticks *Ixodes persulcatus* and their *Borrelia* prevalence at the north-western part of their distribution

**DOI:** 10.1186/s13071-020-04259-z

**Published:** 2020-07-29

**Authors:** Veli-Matti Pakanen, Jani J. Sormunen, Ella Sippola, Donald Blomqvist, Eva R. Kallio

**Affiliations:** 1 Department of Biological and Environmental Sciences, University of Gothenburg, P.O. Box 463, Gothenburg, 40530 Sweden; 2 Ecology and Genetics Research Unit, University of Oulu, P.O. Box 3000, 90014 Oulu, Finland; 3 Biodiversity Unit, University of Turku, 20014 Turku, Finland; 4 Department of Biology, University of Turku, 20014 Turku, Finland; 5 Department of Biological and Environmental Science, University of Jyväskylä, P.O. Box 35, 40041 Jyväskylä, Finland

**Keywords:** Finland, Coastal forest, Co-infection, *Ixodes persulcatus*, Temporal tick dynamics

## Abstract

**Background:**

Because ixodid ticks are vectors of zoonotic pathogens, including *Borrelia*, information of their abundance, seasonal variation in questing behaviour and pathogen prevalence is important for human health. As ticks are invading new areas northwards, information from these new areas are needed. Taiga tick (*Ixodes persulcatus*) populations have been recently found at Bothnian Bay, Finland. We assessed seasonal variation in questing abundance of ticks and their pathogen prevalence in coastal deciduous forests near the city of Oulu (latitudes 64–65°) in 2019.

**Methods:**

We sampled ticks from May until September by cloth dragging 100 meters once a month at eight study sites. We calculated a density index (individuals/100 m^2^) to assess seasonal variation. Samples were screened for *Borrelia burgdorferi* (*sensu lato*) (including *B. afzelii*, *B. garinii*, *B. burgdorferi* (*sensu stricto*) and *B. valaisana*), *Borrelia miyamotoi*, *Anaplasma phagocytophilum*, *Rickettsia* spp., *Neoehrlichia mikurensis*, *Francisella tularensis* and *Bartonell*a spp., *Babesia* spp. and for the tick-borne encephalitis virus.

**Results:**

All except one nymph were identified as *I. persulcatus.* The number of questing adults showed a strong peak in May (median: 6.5 adults/100 m^2^), which is among the highest values reported in northern Europe, and potentially indicates a large population size. After May, the number of questing adults declined steadily with few adults still sampled in August. Nymphs were present from May until September. We found a striking prevalence of *Borrelia* spp. in adults (62%) and nymphs (40%), with *B. garinii* (51%) and *B. afzelii* (63%) being the most common species. In addition, we found that 26% of infected adults were coinfected with at least two *Borrelia* genospecies, mainly *B. garinii* and *B. afzelii*, which are associated with different host species.

**Conclusions:**

The coastal forest environments at Bothnian Bay seem to provide favourable environments for *I. persulcatus* and the spread of *Borrelia*. High tick abundance, a low diversity of the host community and similar host use among larvae and nymphs likely explain the high *Borrelia* prevalence and coinfection rate. Research on the infestation of the hosts that quantifies the temporal dynamics of immature life stages would reveal important aspects of pathogen circulation in these tick populations.
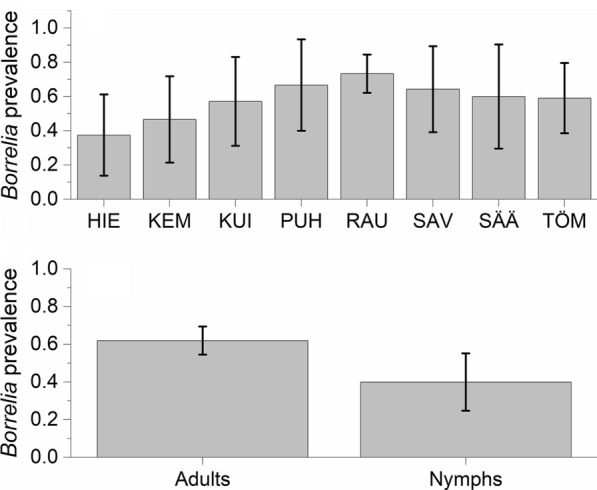

## Background

Zoonotic pathogens transmitted by vectors are increasingly important for human health [1]. In the northern hemisphere, ixodid ticks are the most important vectors for several pathogens of medical and veterinary interest, particularly the TBE-virus (TBEV) and various *Borrelia* species [2–4]. Information on tick population dynamics, seasonal variation in questing behaviour and pathogen prevalence are therefore important for public health. In northern Eurasia, *Ixodes* ticks have been described to have mainly unimodal or bimodal seasonal patterns around the warmer months of the year [5–9]. However, as their abundance and questing behaviour are dependent on multiple environmental factors [9–11], it is difficult to predict seasonal dynamics across the vast ranges of these species - especially when species’ distributions change.

Global warming due to climate change has benefited ixodid species as winters have become more benign and tick activity periods have become longer and warmer in the north [12–15], the effects of which are evident for example in altitudinal studies [6]. Consequently, tick populations have increased, and their distributions have shifted northwards [14, 16, 17]. As ticks invade new areas and face different environments, their abundance and seasonal questing activity may be different from those in the core areas [5, 10], warranting also localized studies.

One of the main vectors of *Borrelia* in Eurasia is the taiga tick, *Ixodes persulcatus*, see [18]. Its distribution currently extends from Fennoscandia to Japan [3, 18]. The taiga tick has been identified in Fennoscandia only recently [19, 20], and this species has been reported to have invaded new areas west- and northwards in northern Europe, up to north-western Finland and eastern Sweden [15, 17]. However, their abundance and seasonal questing behaviour in these newly discovered areas of *I. persulcatus* establishment have not been examined with standard methods in Finland (but see Laaksonen et al. [17] for a seasonal pattern of ticks collected *via* a citizen science campaign in Finland). Questing *I. persulcatus* typically show a rapidly increasing peak in the spring that fades quickly [18], but some seasonal variation can occur [5].

We studied *I. persulcatus* at the north-western part of their distribution range at Bothnian Bay, Finland (between latitudes 64–65°), with the standard cloth dragging method. We examined (i) the abundance of questing *I. persulcatus* using a density index, (ii) seasonal variation in questing activity, and (iii) prevalence of a suite of pathogens. We concentrated on coastal deciduous forests because these coastal areas are known to harbour *I. persulcatus* [15, 17], and because the host communities are often habitat specific causing spatial variation in tick abundance and pathogen prevalence [8, 21–23].

## Methods

**Fig. 1:**
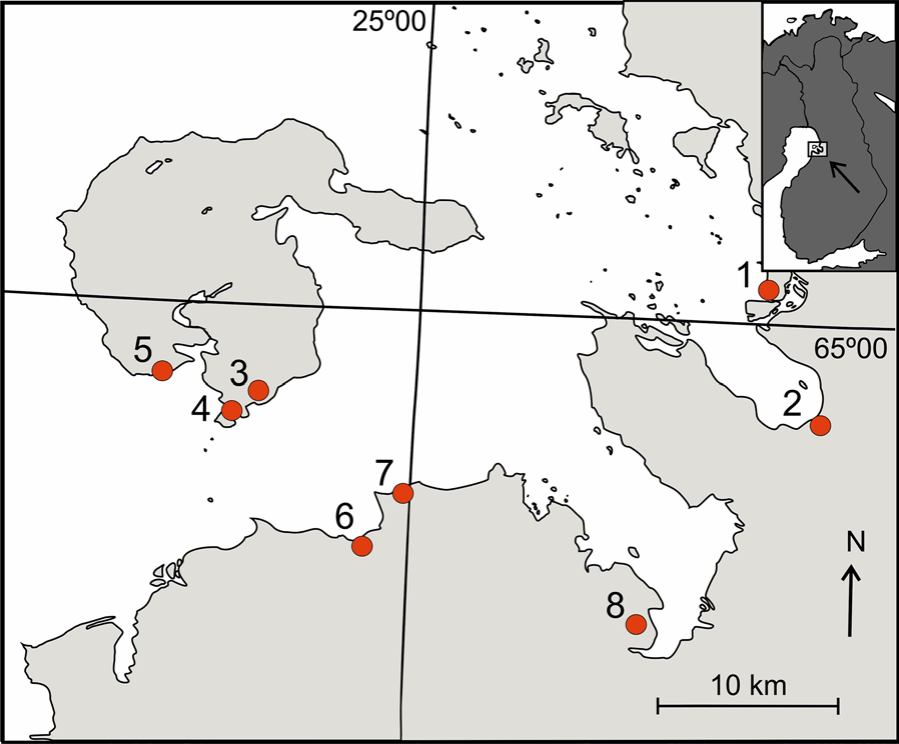
Location of sampling sites at Bothnian Bay, Finland. *Key*: 1, Hietasaari, Oulu; 2, Kempeenlahti, Oulu; 3, Tömppä, Hailuoto; 4, Rautaletto, Hailuoto; 5, Kuivasäikkä, Hailuoto; 6, Savilahti, Siikajoki; 7, Säärenperä, Siikajoki; 8, Puhkiavanperä, Lumijoki)

**Fig. 2:**
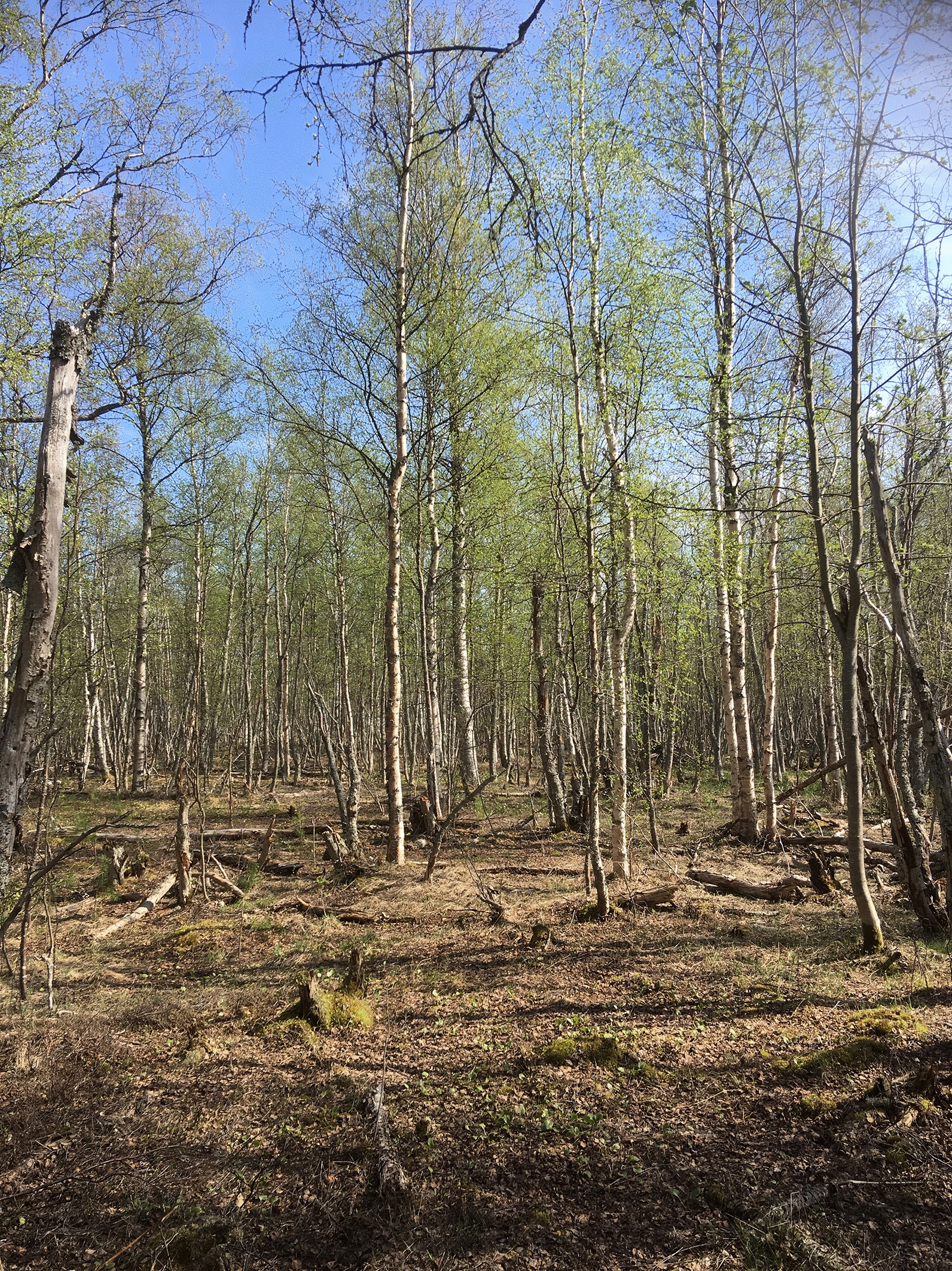
Sampling sites were characterised by birch trees with varying amounts of undergrowth. (Kuivasäikkä, Hailuoto, 10th May 2019)

We examined seasonal variation in questing tick abundance at 8 sites on the coast of Bothnian Bay in Finland (65°01′–64°52′N; 24°41′–25°29′E; Fig. 1). These sites were chosen on the basis of earlier information on the existence of ticks (VMP, unpublished observations). The study sites were early successional deciduous forests (Fig. 2), with willows (*Salix* sp.), reedbed (*Phragmites australis*) and coastal meadows or pastures separating them from the shoreline. The distance to the shoreline varied from 180 m to 900 m. The forests included mainly birch (*Betula pendula*) and alder (*Alnus incana*), but also some European bird cherry (*Prunus padus*), willows (*Salix* spp.) and rowan (*Sorbus aucuparia*). Undergrowth included e.g. dwarf cornel (*Cornus suecica*), arctic bramble (*Rubus arcticus*) and different hay species. These coastal areas are very low and flat, and the coastal forests are characterized by standing water tables in early May to June.

Because these areas are covered by snow until late April, we started sampling in May. Each site was sampled once a month. We used the standard cloth dragging method to examine an index for tick density [24]. We dragged a 1 m × 1 m white flannel flag that was connected to a plastic tube. Each sampling occasion included dragging for 100 m on the surface of vegetation. We stopped every 10–15 m to collect ticks into 1.5 ml plastic tubes with 70% ethanol. Each month, we sampled slightly different transects within the same forest fragment. Dragging was done only when it had not been raining on that day and the vegetation was dry. We dragged during the day between 10:00 h and 18:00 h. Air temperature at the time of sampling varied between 9–23 °C. Collected ticks were identified to the species level using morphological keys [25] and/or a duplex qPCR assay targeting the ITS2 region of *I. persulcatus* and *I. ricinus*, as described previously [26].

### Pathogen analysis

Total DNA and RNA was extracted from tick samples using NucleoSpin® RNA kits and RNA/DNA buffer sets (Macherey-Nagel, Düren Germany), following the kit protocols (NucleoSpin 96 RNA Core Kit: Rev. 05/April 2014 and RNA/DNA buffer set: Rev. 09/April 2017). RNA extracts were stored at − 80 °C for later analyses. DNA extracts were stored at − 20 °C. In addition to the ticks collected from study sites, we also included 7 adults that were collected from an area close to Tömppä, Hailuoto in the analyses.

DNA samples were screened for bacterial pathogens *Borrelia burgdorferi* (*s.l*.) (including specific analyses for *B. afzelii*, *B. garinii*, *B. burgdorferi* (*s.s*.) and *B. valaisana*), *Borrelia miyamotoi*, *Anaplasma phagocytophilum*, *Rickettsia* spp., *Neoehrlichia mikurensis*, *Francisella tularensis* and *Bartonella* spp., and for protozoan parasites *Babesia* spp. Furthermore, RNA samples were screened for tick-borne encephalitis virus (TBEV). The primers used for each pathogen are provided in Additional file 1: Tables S1 and S2.

Real-time quantitative PCR (henceforth abbreviated qPCR) assays were carried out using SensiFAST™ Probe Lo-ROX Kit (for DNA) and SensiFAST™ Probe Lo-ROX One-Step Kit (for RNA) (Bioline, Luckenwalde, Germany). All DNA/RNA samples were analyzed in two replicate reactions carried out on 96 or 384-well plates. At least two non-template negative controls (template replaced with distilled water) were used in each assay. The positive controls used are provided in Additional file 1: Text S1. Samples were considered positive when successful amplification was detected in both replicate reactions or in 2 consecutive assays. Assay protocols are reported in the Additional file 1: Text S1.

Samples found positive for *Rickettsia* by qPCR were subsequently amplified by conventional PCR and Sanger sequenced in order to determine species (Additional file 1: Table S1). Likewise, some *B. burgdorferi* (*s.l*.) positive samples that could not be identified to the genospecies level or that gave unconventional signals in qPCR were Sanger sequenced to determine species (Additional file 1: Table S1). Assay protocols and mastermix contents for PCR amplification were as reported previously (for *Borrelia*: [8]; for *Rickettsia*: [22]), with the following modifications regarding *Borrelia*: the reaction volume was increased to 15 µl, with 3 µl DNA template, and the thermal cycling profile was run for 50 cycles with an annealing temperature of 54 °C.

### Statistical analysis

We used a generalised linear mixed model (GLMM, binomial errors, logit link) to examine the probability of *Borrelia* infection (tick infected or not by *Borrelia burgdorferi* (*s.l*.) between stages (nymphs vs adults) and linear temporal change across the months from May until September by including the sampling site (8 sites) as a random factor. The model was ran using function *‘glmer’* in package *lme4* [27] in R version 3.6.1. [28].

## Results

### Seasonal variation in the density index

**Fig. 3:**
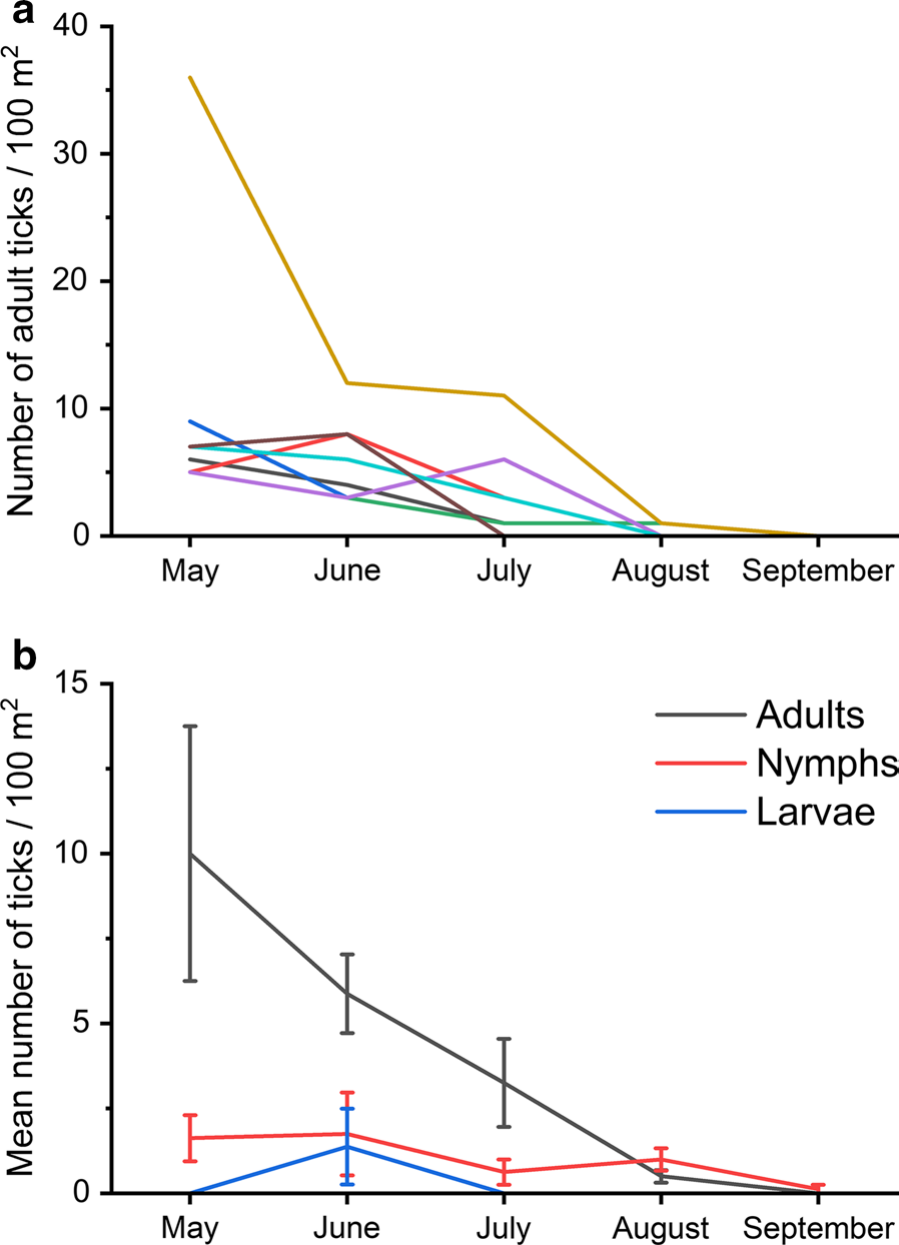
Seasonal variation in abundance of questing *I. persulcatus* (individuals/100 m^2^) in coastal deciduous forests at Bothnian Bay, Finland in 2019. **a** Adults separately for each of the eight sites. **b** Mean ± standard error (SE) for adults, nymphs and larvae (see Additional file 2: Table S3 for data)

We flagged 4.0 km of transect (8 transects, 100 m^2^ per month from May until September) and collected altogether 207 *I. persulcatus* ticks (79 females, 78 males, 40 nymphs and 10 larvae; Additional file 2: Table S3), the ratio being 15.6/4/1 between adults/nymphs/larvae. There was a clear peak in abundance in May, during which densities ranged between 5 and 36 adults per 100 m^2^, with a median of 6.5 (average of 10.0 ± 3.6 SE) (Fig. 3a, b). After May, their numbers gradually declined until August. The observed activity period of adults lasted 101 days from the 1st May until 9th August. Nymphs were less numerous but their observed activity season lasted 120 days from 10th May until 6th September (Fig. 3a). We observed larvae only in June at 2 sites (Fig. 3b). One of the nymphs collected from Rautaletto, Hailuoto was identified as *I. ricinus* and was removed from further analyses.

### Pathogens

**Fig. 4:**
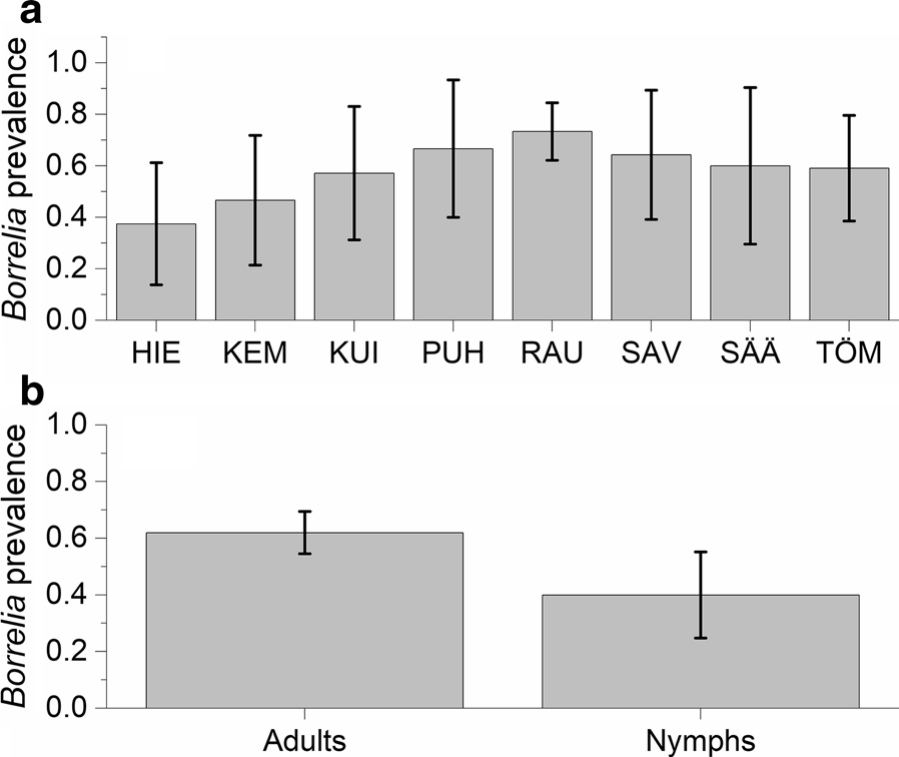
*Borrelia* prevalence (± 95 CI) of questing *I. persulcatus* in coastal deciduous forests at Bothnian Bay, Finland in 2019. **a** Among adults separately for each of the eight sites. **b** In general for adults and nymphs. See Fig. 1 for site locations. *Abbreviations*: HIE, Hietasaari; KEM, Kempeleenlahti; KUI, Kuivasäikkä; PUH, Puhkiavanperä; RAU, Rautaletto; SAV, Savilahti; SÄÄ, Säärenperä; TÖM, Tömppä

The prevalence of *B. burgdorferi* (*s.l*.) was 62% (95% CI: 55–70%) out of 163 sampled adults (males 65%, 95% CI: 54–76%, *n* = 79; females 60%, 95% CI: 50–70%, *n* = 84; Additional file 3: Table S4, Additional file 4: Table S5). There was site specific variation in infection rates with Rautaletto in the island of Hailuoto having highest infection rates (Fig. 4a). Out of 40 nymphs, 40% (95% CI: 25–55%) were infected with *Borrelia* spp. However, no statistically significant differences in *Borrelia* infection probability between adults and nymphs were found after accounting for variation within months and sites (Table 1, Fig. 4b). Prevalence did not show a linear change across the season (Table 1). None of the larvae (*n* = 9) were infected.

**Table 1:** Generalised linear mixed model examining the probability of *Borrelia* spp. infection in *I. persulcatus* individuals

Variable	Coefficient	SE	*Z*-value	*P*-value
Intercept	0.114	1.103	0.104	0.917
Stage (adult)	0.742	0.395	1.878	0.060
Month	− 0.087	0.166	0.522	0.601
Random				
Site	Variance: 0.160	*n* = 8		
No. of observations	203			

*Notes*: Results from a generalised linear mixed model (binomial errors, logit link) examining the effects of developmental stage and linear change with month and including site as a random factor (see Fig. 1) on probability of *Borrelia* infection in *I. persulcatus* individuals

The most common *B. burgdorferi* (*s.l*.) genospecies were *B. garinii* (51% of infected adults) and *B. afzelii* (63% of infected adults), while prevalence was low for *B burgdorferi* (*s.s*.) (7%). Only 4% of samples included *B*. *miyamotoi*, whereas *B. valaisiana* was not detected. The distribution of these *Borrelia* species was similar among infected nymphs (*n* = 16; *B. garinii*: 38%; *B. afzelii*: 50%; *B. burgdorferi* (*s.s*.): 6%; *B. miyamotoi*: 6%). Mixed infections occurred in 26 adults (26% of all infected individuals) but only one (6%) was found among nymphs (Additional file 4: Table S5). These mostly consisted of co-infections between *B. garinii* and *B. afzelii* (19 samples). In addition to these, three samples with *B. garinii* and *B. burgdorferi* (*s.s*.), three with *B. afzelii* and *B. miyamotoi*, one *with B. afzelii* and *B. burgdorferi* (*s.s*.) and one with *B. garinii* and *B. miyamotoi* were detected.

Partial sequencing of the *flagellin* gene for a subset of *B. burgdorferi* (*s.l*.) positive samples revealed four samples of *B. garinii* that differed from each other by 3–8 bp over the 355 bp product, but were 100% identical to different *B. garinii* sequences reported from Russia, Japan and China (e.g. one sample with GenBank reference samples LT631698.1, KU672558.1 and CP003151.1). These sequences also differed by 8–10 bp from concurrently sequenced *B. garinii* obtained from *I. ricinus* nymphs from Helsinki in southern Finland (Additional file 5: Table S6). In addition, a further three samples from the Bothnian Bay coast with identical sequences were highly similar to two reference *B. bavariensis* sequences (Additional file 5: Table S6). *Borrelia afzelii* sequences from the Bothnian Bay (2 unique sequences) were identical to sequences from *I. ricinus* in Helsinki (12 unique sequences) and a reference sample (GenBank: CP009058.1).

Two adults were infected by *A. phagocytophilum* and six adults were infected with *Rickettsia* (5/6 with *R. tarasevichiae* and 1/6 with *R. helvetica*). We found no samples positive for TBEV, *Bartonella* spp., *Babesia* spp., *F. tularensis* or *N. mikurensis*.

## Discussion

Questing *I. persulcatus* showed unimodal seasonal questing behaviour in coastal deciduous forests in the north-western part of its range in northern Finland, with an activity period lasting for at least 101 days. A strong peak in abundance was observed in May, which was followed by a gradual decline, with questing adults still present at low (0.5 individuals/100 m^2^) densities until August but absent in September. Similar activity patterns have been observed in previous studies concerning *I. persulcatus* populations from the species core distribution in Russia [18, 29], but in our study the observed peak was higher and the length of the season longer than expected in these northern conditions [5]. However, the length of the questing season may also reflect variation in host availability and the start of behavioural diapause [30]. Questing was most likely initiated in late April after the snow melted [18]. Thus, the risk of tick bites was high starting from late April until June. Importantly, there was also strong geographical variation in questing activity, as some areas also had higher numbers of questing adults in July (Fig. 3a).

The peak density index (median 6.5 and mean 10 ticks per 100 m^2^) of adults was among the highest reported for *I. persulcatus* in northern Europe (Table 2). Recent studies from Karelia and western Siberia are closest to the median values observed in our study (Karelia: median 3.5 adults/100 m^2^, [11]; western Siberia: median 2.7 adults/100 m^2^, [31]). However, tick densities can show strong spatial variation, as indicated by the particularly high density of *I. persulcatus* observed on an island nearby at northern Bothnian Bay (75 adults/100 m^2^; [32]). One of our study sites also displayed a higher tick density than other sites, with 36 adults/100 m^2^ in May. The densities of adult *I. persulcatus* observed at these sites in the Bothnian Bay are closer to values reported from the eastern parts of the species’ range [33], but comparison can be difficult due to different methodologies.

**Table 2:** Reported densities of adult *Ixodes persulcatus* ticks during their peak occurrence in Europe and Siberia

Site	Lat	Long	Median	Mean	Range	*n*^a^	km^b^	Habitat	Years	Reference
Bothnian Bay, Finland	65°00	25°00	6.5	10.0	5.0–36.0	8	0.8	Decid. forest	2019	This study
Eastern Finland	62°40	31°00	–	0.02^d^	0.0–1.1	96	235	Forest	2008–2009	Bugmyrin et al. [49]
Norrbotten, Sweden	65°44	23°46	0.7^d^	12.7^d^	0.0–75.3	7	2.1	Mixed forest	2015–2016	Jaenson & Wilhemsson [32]
Karelia, Russia	62°07	33°96	0.6^c,d,e^	0.9^c,d,e^	0.2–2.5 ^c,d,e^	2	–	Forest	1982–1990	Bugmyrin et al. [11]
Karelia, Russia	62°07	33°96	3.5 ^c,d,e^	4.0^c,d,e^	2.1–9.1^c,d,e^	9	2.5–18.9	Various	1995–2017	Bugmyrin et al. [11]
Karelia, Russia	61°50	33°12	2.4^d^	2.0^d^	1.1–2.6	3	138.2	Forest	2006–2010	Bugmyrin et al. [50]
Karelia, Russia	61°26	33°16	1.2^d^	1.3^d^	0.2–4.4	14	109.1	Forest	2006–2010	Bugmyrin et al. [50]
Karelia, Russia	62°12	33°50	0.7^d^	0.7^d^	0.0–1.9	10	119.0	Forest	2006–2010	Bugmyrin et al. [50]
Western Siberia, Russia	56°20	84°57	2.7^c^	3.8^c^	1.7–11.8^c^	1	*c.*8	Forest	2006–2013	Romanenko and Leonovich [31]

^a^The number of sampled sites

^b^Length of the dragged distance in km

^c^Values are calculated from yearly data

^d^Peak was not distinguished with seasonal cloth dragging

^e^Values were approximated from Fig. 1 in Bugmyrin et al. [11]

*Note*: Mean and median number of adult *Ixodes persulcatus* ticks collected per 100 m^2^ during their peak occurrence in Northern Europe and Siberia

While we acknowledge that the tick density estimates obtained by cloth dragging do not give precise approximations of tick population size, the relatively high density-index found in this study that suggests a thriving population is somewhat surprising. This is because the general environmental conditions in the Oulu region are suboptimal in terms of the growing season (155–165 days; https://ilmatieteenlaitos.fi/), cumulative temperature sum is lower (1100–1200 °C) and precipitation (260–320  mm per year), compared to those suggested for taiga ticks [10]. The high density-index may therefore reflect more favourable conditions in the coastal forests. Indeed, *I. persulcatus* seem to be less abundant further from the coast in northern Finland [17]. At least, the main hosts of adult *I. persulcatus*, cervids [18] such as moose (*Alces alces*) and roe deer (*Capreolus capreolus*) are relatively common in this region.

The high density-index of questing *I. persulcatus* adults is concurrent with a longer residence at the coast of the Bothnian Bay than currently known. Sightings of blood-sucking ticks have been made from the 1930s onwards by people living in Siikajoki (1960s), Lumijoki (1980s), Hailuoto (1930s), Oulu (1970s) and Ii (1970s; [34, 35], Juha Markkola and Jari Ylönen personal communication). An early examination of tick distribution in Finland in the 1950s, executed as a questionnaire to veterinarians, did not find ticks in this region [36]. It is possible that ticks have been low in abundance during these periods at northern Ostrobothnia and perhaps located mostly within the coastal forests of the Bothnian Bay. However, given the current distribution of *I. persulcatus* and *I. ricinus* [17], it is perhaps more likely that these old observations were *I. persulcatus* rather than *I. ricinus*. Thus, the recent findings might not reflect recent expansion to this region of the Bothnian Bay, but rather an increase in abundance [19, 20, 37]).

The number of adults was four times that of nymphs, reflecting the limited applicability of cloth dragging in measuring abundance of immature stages of *I. persulcatus* [18, 33]. Indeed, as opposed to *I. ricinus* nymphs that are also more easily collected by dragging, *I. persulcatus* nymphs rarely feed on larger hosts such as humans. Our results nonetheless revealed that the questing season of nymphs was longer than that of the adults, extending until September with a minimum activity period of 120 days.

We found a striking prevalence of *Borrelia* in *I. persulcatus* across our study sites: 62% of adults and 40% of nymphs carried *Borrelia*. These values concur with the generally higher infection rate of *I. persulcatus* compared to *I. ricinus*, for which *Borrelia* prevalence usually varies between 0–45% [38–40]. Naturally, *Borrelia* prevalence is lower in large scale studies that include a variety of environments. For example, a citizen science study, which sampled ticks across Finland, found *Borrelia* prevalence of about 22% in adult *I. persulcatus* [4]. Our results from coastal forests therefore stand out significantly. Similar high *Borrelia* prevalence (up to 69%) has been described among *I. persulcatus* for some specific localities in larch and larch-birch forests in Russia [41]. Interestingly, a recent study of *I. persulcatus* populations in the Swedish islands at the Bothnian Bay also reported high *Borrelia* prevalence (55%; [32]).

High infections rates can be expected among *I. persulcatus*, as both immature stages feed on small host species that are reservoirs of *Borrelia* [18]. Similar proportions of *B. garinii* and *B. afzelii* infections among adult ticks suggests that both birds and small mammals act as hosts of the immature stages [32]. Such feeding patterns give a plausible explanation for the relatively high coinfection rate of 26% among *Borrelia-*infected adult ticks that occurred mainly with *B. garinii* and *B. afzelii*. Coinfection rates of these genospecies varies (e.g. [42, 43]). Other explanations for coinfections include interrupted feeding [44] and transmission of *Borrelia* through co-feeding on hosts [45]. Importantly, we also found a low diversity of other tick-borne pathogens. This is consistent with results from the Swedish side of the Bothnian Bay and may indicate a low diversity of host species for the larval and nymph stages [32]. A low host diversity may increase the overall infection rate among adult ticks [46]. These intriguing results warrant further studies on the feeding patterns of *I. persulcatus* on different hosts and on the *Borrelia* species infecting their hosts.

Interestingly, flagellin amplicons from a subset of *B. burgdorferi* (*s.l*.) positive samples revealed conserved *B. afzelii* sequences but varying *B. garinii* sequences, both within the coastal areas of the Bothnian Bay as well as between different areas in Finland. While none of the *B. garinii* sequences obtained from *I. persulcatus* were identical, they displayed 100% identity matches with various sequences reported from Russia, Japan and China, which consequently also appear to mostly originate from *I. persulcatus*. This suggests the possible presence of an eastern and/or *I. persulcatus* associated strain (or strains). In contrast, nine identical *B. garinii* sequences were attained from nine *I. ricinus* nymphs collected from different areas in Helsinki. It remains to be determined whether the apparent existence of different *B. garinii*/*B. garinii*-like strains in the Bothnian Bay coast and Helsinki is a consequence of some tick species specific factor or, for example, geographical variation in available host animal species. Whatever the cause, these observations are in line with recently reported observations from across Europe, which revealed that little geographical structuring within *B. garinii* strains can be detected [47]. This is expected to be at least partly on account of their highly mobile avian reservoir hosts, which may effectively and quickly transfer different strains across vast geographical ranges.

In addition, a further three sequences from the Bothnian Bay were found to be identical with each other and highly similar to two reference samples of *B. bavariensis*. *Borrelia bavariensis* is a genospecies closely associated with *B. garinii*, which has not been reported from Finland thus far [48]. Unfortunately, due to their close likeness, the reliable differentiation of *B. bavariensis* and *B. garinii* requires more thorough molecular methods, such as multilocus sequence analysis [48]. Consequently, the possible presence of *B. bavariensis* samples reported as *B. garinii* in GenBank also cannot be discounted, further complicating the identification of the genospecies, particularly based on sequences of a single target gene. As such, the precise identity of these three *B. bavariensis*-like samples remains undetermined. In any case, the occurrence of these different *B. garinii*/*B. bavariensis*-like bacteria at the Bothnian Bay may explain why the genospecies-specific qPCR analyses gave missing or abnormal signals in their case.

## Conclusions

We show a relatively high peak abundance of questing *I. persulcatus* in May at the north-western part of their distribution, along with gradually declining questing activity towards the autumn. Overall, the activity period of *I. persulcatus* lasted until August among adults and until September among nymphs. Furthermore, this *I. persulcatus* population had a very high prevalence of *Borrelia* but low diversity of other pathogens. Taken together, our results suggest that these coastal forest environments may provide favourable environments for reproduction and survival of *I. persulcatus*, and the spread of *Borrelia*.

## Supplementary information

**Additional file 1: Text S1.** Additional laboratory protocols. **Table S1.** Primers and probes used in tick-borne pathogen screening and sequencing. **Table S2.** Mastermix contents for qPCR analyses of tick-borne pathogens.**: Text S1.** Additional laboratory protocols. **Table S1.** Primers and probes used in tick-borne pathogen screening and sequencing. **Table S2.** Mastermix contents for qPCR analyses of tick-borne pathogens.

**Additional file 2: Table S3.** Data on the number collected ticks per 100 m^2^. Information sampling days and sites with coordinates and the stage of collected ticks and the sex of adults.

**Additional file 3: Table S4.** Data on pathogen infections for each individual.

**Additional file 4: Table S5.** Data on pathogen infections summarised per area and stage (adults *vs* nymphs).

**Additional file 5: Table S6.** Comparative analysis of *Borrelia* flagellin sequences from I. persulcatus adults from the Bothnian Bay coast (samples labeled BothnianB), *I. ricinus* nymphs from Helsinki (Helsinki) and reference sequences downloaded from GenBank (with accession numbers). Numbers in brackets signify the count of unique, identical sequences. Geneious version 2020.0 created by Biomatters. Available from https://www.geneious.com.

## Data Availability

All data generated or analysed during this study are included in this published article and its additional files. Representative sequences were deposited in the GenBank database.

## Abbreviations

TBEV: The tick-borne encephalitis virus
GLMM: Generalized linear mixed model
