# Supplementary material for: Questing abundance of adult taiga ticks Ixodes persulcatus and their Borrelia prevalence at the north-western part of their distribution
Source: Parasit Vectors. 2020 Jul 29;13:384. doi: 10.1186/s13071-020-04259-z (PMC7391513; doi:10.1186/s13071-020-04259-z)
Supplement: Supplementary file 1 — Additional file 1: Text S1. Additional laboratory protocols. Table S1. Primers and probes used in tick-borne pathogen screening and sequencing. Table S2. Mastermix contents for qPCR analyses of tick-borne pathogens.: Text S1. Additional laboratory protocols. Table S1. Primers and probes used in tick-borne pathogen screening and sequencing. Table S2. Mastermix contents for qPCR analyses of tick-borne pathogens. [file 13071_2020_4259_MOESM1_ESM.docx]

**Questing abundance of adult taiga ticks *Ixodes persulcatus* and their *Borrelia* prevalence at the north-western part of their distribution**

Technical appendix: Additional protocols and results for laboratory and statistical analyses

**TEXT S1: Additional laboratory protocols**

The primers and probes used in the analyses of tick-borne pathogens are reported in Table S1 and mastermix contents in Table S2. The thermal cycling profile used for qPCR analyses of all pathogens apart from TBEV was 95°C for 5 minutes, then 50 cycles of 95°C for 10 sec and 60°C (58°C for runs with *Rickettsia*) for 30 sec. For TBEV, the thermal cycling profile was 48°C for 10 minutes (reverse transcription) and 95°C for 2 minutes, then 50 cycles of 95°C for 5 sec and 60°C for 30 sec.

Laboratory-grown strains of *B. afzelii*, *B. garinii* and *B. burgdorferi* s.s. were used as positive controls for the respective pathogens, whereas for *B. valaisiana* and *B. miyamotoi*, we used samples previously identified via sequencing. Multiple TBEV RNA strains were used as positive controls in TBEV analyses. Patient strains of *Bartonella grahamii* and *B. quintana* (provided by Arto Pulliainen, University of Turku) were used as positive controls for *Bartonella*, whereas for *Rickettsia,* we used a commercially available control (ref. MBC042; Vircell, Granada, Spain). Finally, for *N. mikurensis*, *F. tularensis*, *A. phagocytophilum* and *Babesia,* we used samples previously identified by sequencing.

Table S1. Primers and probes used in tick-borne pathogen screening and sequencing.

| Primer/probe name | Primer/probe target | 5’ 🡪 3’ | Reference |
| --- | --- | --- | --- |
| **qPCR:** |  |  |  |
| Bb23Sf | *B. burgdorferi* 23S RNA | CGAGTCTTAAAAGGGCGATTTAGT | Courtney et al. 2004 |
| Bb23Sr | *B. burgdorferi* 23S RNA | GCTTCAGCCTGGCCATAAATAG |  |
| Bb23Sp | *B. burgdorferi* 23S RNA | [FAM]-AGATGTGGTAGACCCGAAGCCGAGTG-[BHQ1] |  |
|  |  |  |  |
| Baf-RecA-F | *B. afzelii recA* | AGTCAGCCTGATACCGGAGA | Klemola et al. 2019 |
| Baf-RecA-R | *B. afzelii recA* | ATTTTGGGGTCAAAGCTGCC |  |
| Baf-RecA-P | *B. afzelii recA* | [FAM]-TGCCGAACATTTAATTAGAAG-[BHQ1] | Tventen et al. 2013 |
|  |  |  |  |
| Bga-RecA-F | *B. garinii recA* | ATGCAAAAGCTTTGGGGGTT | Klemola et al. 2019 |
| Bga-RecA-R | *B. garinii recA* | AGGGGTTAAAGCTGCTACAGA |  |
| Bga-RecA-P | *B. garinii recA* | [HEX]-TTGCCGAACATTTAATCAGAA-[BHQ1] | Tventen et al. 2013 |
|  |  |  |  |
| Bbss-RecA-F | *B. burgdorferi* s.s. *recA* | CCTGATACCGGAGAGCAAGC | Klemola et al. 2019 |
| Bbss-RecA-R | *B. burgdorferi* s.s. *recA* | GGGGTTAAAGCCGCTACAGA |  |
| Bbss-RecA-P | *B. burgdorferi* s.s. *recA* | [HEX]-TTGCTGAGCATTTAATCAGAA-[BHQ1] | Tventen et al. 2013 |
|  |  |  |  |
| Bva-RecA-F | *B. valaisiana recA* | TGGTCCTGAGTCGTCTGGTA | Klemola et al. 2019 |
| Bva-RecA-R | *B. valaisiana recA* | CTTGCTCTCCGGTGTCAGG |  |
| Bva-RecA-P | *B. valaisiana recA* | [Cy5]-AGGTTCAAAAAGAAGGTGGTAT-[BHQ2] | Tventen et al. 2013 |
|  |  |  |  |
| Bmi-F | *B. miyamotoi glpQ* | CACGACCCAGAAATTGACACA | Vayssier-Taussat et al. 2013 |
| Bmi-R | *B. miyamotoi glpQ* | GTGTGAAGTCAGTGGCGTAAT |  |
| Bmi-P | *B. miyamotoi glpQ* | [FAM]-TCGTCCGTTTTCTCTAGCTCGATTGGG-[BHQ1] |  |
|  |  |  |  |
| Bart-ssRA-F | *Bartonella* *ssRa* | GCTATGGTAATAAATGGACAATGAAATAA | Diaz et al. 2012 |
| Bart-ssRA-R | *Bartonella* *ssRa* | GCTTCTGTTGCCAGGTG |  |
| Bart-ssRA-P | *Bartonella ssRa* | [FAM]-ACCCCGCTTAAACCTGCGACG-[BHQ1] |  |
|  |  |  |  |
| Rspp-F | *Rickettsia gltA* | GAGAGAAAATTATATCCAAATGTTGAT | Labruna et al. 2004 |
| Rspp-R | *Rickettsia gltA* | AGGGTCTTCGTGCATTTCTT |  |
| Rspp-P | *Rickettsia gltA* | [CY5]-CATTGTGCCATCCAGCCTACGGT-[BHQ3] |  |
|  |  |  |  |
| CNeGroEL-F | *N. mikurensis groEL* | CCTTGAAAATATAGCAAGATCAGGTAG | Jahfari et al. 2012 |
| CNeGroEL-R | *N. mikurensis groEL* | CCACCACGTAACTTATTTAGCACTAAAG |  |
| CNeGroEL-P | *N. mikurensis groEL* | [FAM]-CCTCTACTAATTATTGCWGAAGATGTAGAAGGTGAAGC-[BHQ1] |  |
|  |  |  |  |
| ApMSP2F | *A.phagocytophilum Msp2* | ATGGAAGGTAGTGTTGGTTATGGTATT | Courtney et al. 2004 |
| ApMSP2R | *A.phagocytophilum Msp2* | TTGGTCTTGAAGCGCTCGTA |  |
| ApMSP2P | *A.phagocytophilum Msp2* | [CY5]-TGGTGCCAGGGTTGAGCTTGAGATTG-[BBQ650] |  |
|  |  |  |  |
| Bab18S-F | *Babesia* 18S rRNA | CAGCTTGACGGTAGGGTATTGG | Radzijevskaja et al. 2008 |
| Bab18S-R | *Babesia* 18S rRNA | TCGAACCCTAATTCCCCGTTA |  |
| Bab18S-P | *Babesia* 18S rRNA | [HEX]-CGAGGCAGCAACGG-[BHQ1] |  |
|  |  |  |  |
| TBE1-F | TBEV non-struct. prot. 5 | GGGCGGTTCTTGTTCTCC | Schwaiger & Cassinotti 2003 |
| TBE1-R | TBEV non-struct. prot. 5 | ACACATCACCTCCTTGTCAGACT |  |
| TBE-Probe-WT | TBEV non-struct. prot. 5 | [HEX]-TGAGCCACCATCACCCAGACACA-[BHQ2] |  |
|  |  |  |  |
| FTu23-F | *F. tularensis* 23Kda | TGAGATGATAACAAGACAACAGGTAAC | Skottman et al. 2007 |
| FTu23-R | *F. tularensis* 23Kda | GGATGAGATCCTATACATGCAGTAGGA |  |
| FTu23-P2 | *F. tularensis* 23Kda | [HEX]-CCATTCATGTGAGAACTG-[BHQ1] |  |
|  |  |  |  |
| **PCR:** |  |  |  |
| CS877f | *Rickettsia gltA* | GGGGACCTGCTCACGGCGG | Mediannikov et al. 2004 |
| CS1258r | *Rickettsia gltA* | ATTGCAAAAAGTACAGTGAACA |  |
|  |  |  |  |
| BbFLA-F | *B. burgdorferi flagellin* | AGAGCAACTTACAGACGAAATTAAT | Skotarczak et al. 2002 |
| BbFLA-R | *B. burgdorferi flagellin* | CAAGTCTATTTTGGAAAGCACCTAA |  |

| qPCR target | Single/  duplex/  multiplex | Sample(s) | Volume | Probe mix | Forward/reverse primer concentration | Probe concentration | DNA/RNA template |
| --- | --- | --- | --- | --- | --- | --- | --- |
| *B. burgdorferi* s.l. | Single | Single | 5 µl | 2.5 µl | 200 nM | 100 nM | 1 µl |
| *Rickettsia*  *Bartonella* spp.  *F.tularensis* | Multiplex  Multiplex  Multiplex | Single | 8 µl | 4 µl | 300 nM  200 nM  300nM | 150 nM  100 nM  150nM | 1 µl |
| *A. phagocytophilum*  *Babesia* spp.  *N. mikurensis* | Multiplex  Multiplex  Multiplex | Single | 8 µl | 4 µl | 400 nM  400 nM  200 nM | 200 nM  200 nM  100 nM | 1 µl |
| TBEV | Single | Single | 5 µl | 2,5 µl | 400 nM | 100 nM | 1,9 µl |
| *B. afzelii*  *B. burgdorferi* s.s.  *B. valaisiana* | Multiplex  Multiplex  Multiplex | Single | 8 µl | 4 µl | 300 nM | 150 nM | 2 µl |
| *B. garinii*  *B. miyamotoi* | Duplex  Duplex | Single | 8 µl | 4 µl | 300 nM  200 nM | 150 nM  100 nM | 2 µl |

Table S2. Mastermix contents for qPCR analyses of tick-borne pathogens.

**References:**

Courtney JW, Kostelnik LM, Zeidner NS, Massung RF. Multiplex Real-Time PCR for Detection of Anaplasma phagocytophilum and Borrelia burgdorferi. J Clin Microbiol. 2004;42(7):3164-8.

Diaz MH, Bai Y, Malania L, Winchell JM, Kosoy MY. Development of a novel genus- specific real- time PCR assay for detection and differentiation of Bartonella species and genotypes. J Clin Microbiol. 2012;50.

Jahfari S, Fonville M, Hengeveld P, Reusken C, Scholte E-J, Takken W, et al. Prevalence of Neoehrlichia mikurensis in ticks and rodents from North-west Europe. Parasit Vector. 2012;5(1):74

Klemola T, Sormunen JJ, Mojzer J, Mäkelä S, Vesterinen EJ. High tick abundance and diversity of tick-borne pathogens in a Finnish city. Urb. Ecosyst. 2019; 1-10.

Labruna MB, Whitworth T, Horta MC, Bouyer DH, McBride JW, Pinter A. Rickettsia species infecting Amblyomma cooperi ticks from an area in the State of São Paulo, Brazil, where Brazilian spotted fever is endemic. J Clin Microbiol. 2004;42.

Mediannikov OY, Sidelnikov Y, Ivanov L, Mokretsova E, Fournier PE, Tarasevich I. Acute tick-borne rickettsiosis caused by Rickettsia heilongjiangensis in Russian Far East. Emerg Infect Dis. 2004;10.

Radzijevskaja J, Paulauskas A, Rosef O. Prevalence of Anaplasma phagocytophilum and Babesia divergens in Ixodes ricinus ticks from Lithuania and Norway. Int J Med Microbiol. 2008;298:218-21.

Schwaiger M, Cassinotti P. Development of a quantitative real-time RT-PCR assay with internal control for the laboratory detection of tick borne encephalitis virus (TBEV) RNA. J Clin Virol. 2003;27(2):136-45.

Skotarczak B, Wodecka B, Cichocka A. Coexistence DNA of *Borrelia burgdorferi* sensu lato and *Babesia microti* in *Ixodes ricinus* ticks from North-western Poland. Ann Agric Environ Med. 2002;9(1):25-28.

Skottman T, Piiparinen H, Hyytiäinen H, Myllys V, Skurnik M, Nikkari S. Simultaneous real-time PCR detection of *Bacillus anthracis*, *Francisella tularensis* and *Yersinia pestis*. Eur J Clin Microbiol Infect Dis. 2007; 26(3):207-211.

Tventen AK. Prevalence of *Borrelia burgdorferi* sensu stricto, *Borrelia afzelii*, *Borrelia garinii*, and *Borrelia valaisiana* in *Ixodes ricinus* ticks from the northwest of Norway. Scand J Infect Dis. 2013;45(9):681-687.

Vayssier-Taussat M, Moutailler S, Michelet L, Devillers E, Bonnet S, Cheval J, et al. Next Generation Sequencing Uncovers Unexpected Bacterial Pathogens in Ticks in Western Europe. PLoS ONE. 2013; e81439.
