# Supplementary material for: Questing abundance of adult taiga ticks Ixodes persulcatus and their Borrelia prevalence at the north-western part of their distribution
Source: Parasit Vectors. 2020 Jul 29;13:384. doi: 10.1186/s13071-020-04259-z (PMC7391513; doi:10.1186/s13071-020-04259-z)
Supplement: Supplementary file 4 — Additional file 4: Table S5. Data on pathogen infections summarised per area and stage (adults vs nymphs). [file 13071_2020_4259_MOESM4_ESM.docx]

**Questing abundance of adult taiga ticks *Ixodes persulcatus* and their *Borrelia* prevalence at the north-western part of their distribution**

Table S5 Prevalence of different *Borrelia* species (Bmi = *B. miyamotoi*, Bbss = *burgdorferi s.s.:* Bga= *garinii*; Baf = *afzelii*), Rspp = *Ricketssia* and Ana = *Anaplasma* in *Ixodes persulcatus* ticks collected by flagging vegetation at Bothnian Bay, Finland separately for adults, nymphs and larvae.

|  |  | *Borrelia* | |  |  |  |  |  | Mixed *Borrelia* | |  |  |
| --- | --- | --- | --- | --- | --- | --- | --- | --- | --- | --- | --- | --- |
| Adults | N | N(Bor) | % | Bmi | Bga | Bbss | Baf | Undef. | N | % | Rspp | Ana |
| Hietasaari | 16 | 6 | 38 |  | 4 |  | 3 |  | 1 | 17 | 0 | 0 |
| Kempeleenlahti | 15 | 7 | 47 |  | 3 |  | 5 |  | 1 | 14 | 0 | 0 |
| Kuivasäikkä | 14 | 8 | 57 | 1 | 4 | 2 | 5 |  | 4 | 50 | 0 | 1 |
| Puhkiavanperä | 12 | 8 | 67 |  | 5 | 2 | 2 | 1 | 2 | 25 | 0 | 0 |
| Rautaletto | 60 | 44 | 73 | 1 | 22 | 2 | 31 | 1 | 12 | 27 | 4 | 1 |
| Savilahti | 14 | 9 | 64 |  | 6 | 1 | 5 |  | 3 | 33 | 0 | 0 |
| Säärenperä | 10 | 6 | 60 |  | 5 |  | 1 |  | 0 | 0 | 0 | 0 |
| Tömppä | 22 | 13 | 59 | 2 | 3 |  | 12 |  | 3 | 21 | 2 | 0 |
| TOTAL | 163 | 101 | 62 | 4 (4%) | 52 (51%) | 7 (7%) | 64 (63%) | 2 (2%) | 26 | 26 | 6 | 2 |
|  |  |  |  |  |  |  |  |  |  |  |  |  |
| Nymphs | N | N(Bor) | % | Bmi | Bga | Bbss | Baf | Undef. | N | % | Rspp | Ana |
| Hietasaari | 2 | 0 | 0 |  |  |  |  |  |  |  | 0 | 0 |
| Kempeleenlahti | 18 | 6 | 33 |  | 2 |  | 3 | 1 | 0 | 0 | 0 | 0 |
| Kuivasäikkä | 3 | 0 | 0 |  |  |  |  |  |  |  | 0 | 0 |
| Puhkiavanperä | 0 | 0 | 0 |  |  |  |  |  |  |  | 0 | 0 |
| Rautaletto | 9 | 7 | 78 | 1 | 3 |  | 4 |  | 1 | 14 | 0 | 0 |
| Savilahti | 3 | 2 | 67 |  | 2 |  |  |  | 0 | 0 | 0 | 0 |
| Säärenperä | 1 | 0 | 0 |  |  |  |  |  |  |  | 0 | 0 |
| Tömppä | 4 | 1 | 25 |  |  |  | 1 |  |  | 0 | 0 | 0 |
| TOTAL | 40 | 16 | 40 | 1 (6%) | 7 (44%) | 1 (6%) | 8 (50%) | 1 (6%) | 1 | 6 | 0 | 0 |
|  |  |  |  |  |  |  |  |  |  |  |  |  |
| Larvae | N | N(Bor) | % | Bmi | Bga | Bbss | Baf | Undef. | double | % | Rspp | Ana |
| Tömppä | 9 | 0 | 0 |  |  |  |  |  |  |  | 0 | 0 |
